# A DNA Metabarcoding Study of a Primate Dietary Diversity and Plasticity across Its Entire Fragmented Range

**DOI:** 10.1371/journal.pone.0058971

**Published:** 2013-03-19

**Authors:** Erwan Quéméré, Fabrice Hibert, Christian Miquel, Emeline Lhuillier, Emmanuel Rasolondraibe, Julie Champeau, Clément Rabarivola, Louis Nusbaumer, Cyrille Chatelain, Laurent Gautier, Patrick Ranirison, Brigitte Crouau-Roy, Pierre Taberlet, Lounès Chikhi

**Affiliations:** 1 Laboratoire « Évolution et Diversité Biologique », CNRS/Université Paul Sabatier/ENFA, Toulouse, France; 2 Laboratoire « Comportement et Écologie de la Faune Sauvage », Institut National de la Recherche Agronomique, Castanet-Tolosan, France; 3 Instituto Gulbenkian de Ciência, Oeiras, Portugal; 4 Laboratoire d’Ecologie Alpine, CNRS/Université Joseph Fourier, Grenoble, France; 5 Faculté des Sciences, Université de Mahajanga, Campus Universitaire Ambondrona, Mahajanga, Madagascar; 6 Laboratoire de botanique systèmatique et biodiversité, Conservatoire et Jardin botaniques de la Ville de Genève and Université de Genève, Chambésy, Switzerland; 7 Département de Biologie et d’Ecologie Végétale, University of Antananarivo, Antananarivo, Madagascar; Natural History Museum of Denmark, University of Copenhagen, Denmark

## Abstract

In tropical regions, most primary ecosystems have been replaced by mosaic landscapes in which species must cope with a large shift in the distribution of their habitat and associated food resources. Primates are particularly vulnerable to habitat modifications. Most species persist in small fragments surrounded by complex human-mediated matrices whose structure and connectivity may strongly influence their dispersal and feeding behavior. Behavioral plasticity appears to be a crucial parameter governing the ability of organisms to exploit the resources offered by new matrix habitats and thus to persist in fragmented habitats. In this study, we were interested in the dietary plasticity of the golden-crowned sifaka (*Propithecus tattersalli*), an endangered species of lemur, found only in the Daraina region in north-eastern Madagascar. We used a DNA-based approach combining the barcoding concept and Illumina next-generation sequencing to (i) describe the species diet across its entire range and (ii) evaluate the influence of landscape heterogeneity on diet diversity and composition. Faeces from 96 individuals were sampled across the entire species range and their contents were analyzed using the *trn*L metabarcoding approach. In parallel, we built a large DNA reference database based on a checklist of the plant species of the Daraina region. Our results suggest that golden-crowned sifakas exhibit remarkable dietary diversity with at least 130 plant species belonging to 80 genera and 49 different families. We highlighted an influence of both habitat type and openness on diet composition suggesting a high flexibility of foraging strategies. Moreover, we observed the presence of numerous cultivated and naturalized plants in the faeces of groups living in forest edge areas. Overall, our findings support our initial expectation that *P. tattersalli* is able to cope with the current level of alteration of the landscape and confirm our previous results on the distribution and the dispersal ability of this species.

## Introduction

The earth is experiencing an accelerated loss of biodiversity. As a consequence, understanding how the landscape structure affects the performance of species and alters the functioning of ecosystems has become a major concern in conservation biology [1]. In the tropics, most primary ecosystems are being replaced by human-dominated mosaic landscapes in which plant and animal populations and species must cope with increasingly large shifts in the distribution of habitat and feeding resources across a range of spatial scales [2], [3]. Primates are particularly vulnerable [4] because almost 90% of all primate species occur in threatened forest tropical ecosystems [5] and one in four species are either Endangered or Critically Endangered [6]. This is particularly true of lemurs that have recently been identified as the most threatened group of mammals (IUCN workshop, Antananarivo 2012). Most primate species persist in small fragments surrounded by complex human-mediated matrices whose composition and connectivity may strongly influence their dispersal, distribution and viability [7], [8], [9]. Species response to habitat alteration may vary widely from population decline or extinction [10], [11] to adaptation and emergence of new behavioral strategies [12]. Previous studies have suggested that simple measures of habitat structure (e.g. fragment size and connectivity, type of vegetation) [13], [14], [15] or species traits (e.g. abundance, home range, body mass, type of diet, etc.) [9], [16] only partly explain this variability of response [17]. Behavioral plasticity appears to be a crucial parameter governing the ability of organisms to exploit the resources offered by new matrix habitats and their subsequent ability to persist in fragmented forests [7], [12]. Therefore, we need to increase our knowledge of species ecological and behavioral flexibility in order to assess their minimum resources requirements at multiple spatial scales (from local spatial scale to landscape scale) [3], [18] and thus improve management and conservation actions.

Diet is one of the primary drivers of primate abundance and demography [19]. It is the basis of critical traits such as body condition or home range size [20] and unavoidably influences activity budget. Adequate nutrition in the face of environmental and social constraints is thus a prerequisite for successful reproduction [21]. Understanding change in foraging strategies over time and across heterogeneous landscape is a promising issue for assessing the degree of flexibility of species in coping with changes in local environmental conditions such as habitat disturbance. However, the studies investigating the intra-specific variability of feeding preferences at the landscape scale are still very limited (but see [17]). Indeed, most authors analyzing the effect of changes in forest composition on primate diet rely on long-term data focusing on only one or few social groups at a local scale (patch scale) [22], [23], [24], [25]. Traditional techniques for determining diets rely on direct observation of foraging or microscopic examination of gut or faeces content. These methods are time consuming and require considerable training for identifying the food items. Furthermore, following animals to identify all the species consumed is extremely difficult for many primate species living in remote and less accessible locations. Exploring diet variability and shifts across multiple spatial scales (from local to landscape scales) is thus particularly challenging. Beyond environmental changes and the associated feeding behavior flexibility of species, it is important to stress that, due to these practical difficulties, little is actually known about the feeding behavior of most species. The emergence of next-generation sequencing (NGS) technologies combined with the massive expansion of animal and plant barcode databases [26], [27] has recently allowed to perform diet analysis by identifying the DNA sequences of consumed items from faecal material, without the need of laborious and expensive steps of cloning of PCR products [28], [29], [30]. These powerful and non-invasive methods can provide accurate dietary profiles for several tens or hundreds of individuals simultaneously and result in extended knowledge on the foraging ecology at a population scale.

In this study, we investigated the dietary diversity and plasticity of the golden-crowned sifaka (*Propithecus tattersalli*), a critically endangered species of lemur, found only in the Daraina region (Loky-Manamabato) in north-eastern Madagascar. The range of this species is among the most restricted of any lemurs [31]. *P. tattersalli* was scientifically described only twenty-five years ago (Simons, 1988) and its spatial ecology has remained poorly studied due to the remoteness of the Daraina region. Some recent studies highlighted a limited influence of habitat fragmentation on its dispersal [32] and the absence of negative edge effect on its distribution within forest fragments [33]. Indeed, family groups (2–10 individuals, average 3.5) were observed in a wide variety of habitats with various levels of openness including agricultural land with crops or small fragments (<100 ha). This suggests that the species may cope with various environmental conditions and resources and has a very plastic response to landscape heterogeneity. The first aim of our study was to describe the diet of golden-crowned sifaka in terms of plant taxa using a DNA-based approach combining barcoding and next-generation sequencing [30] to compare our results with those published by Meyers [34]. We conducted this study in the dry season which, according to Meyers [34], is the period of lowest availability of the sifaka’s preferred food items (seed, fruits, immature leafs). We carried out a comprehensive sampling of sifaka fresh faecal pellets across the entire species range and in parallel built a large DNA barcoding reference database from a checklist of plants of the Daraina region. Our second aim was to use co-inertia analysis to explain the relationship between the spatial patterns of diet diversity and composition and the local environmental heterogeneity, expressed in terms of habitat types and openness.

## Materials and Methods

### Study site, faeces sampling

**Figure 1:**
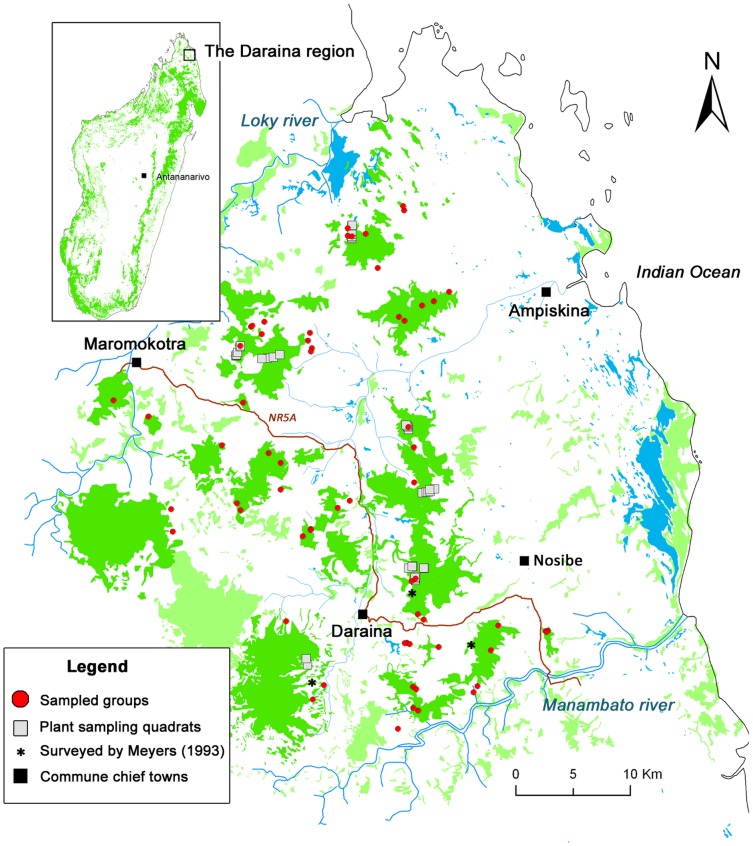
Faeces and plant sampling schemes.

Golden-crowned sifakas inhabit the Daraina region between the Loky and the Manambato rivers in northeastern Madagascar (within the Sava region, **Fig. 1**). Located at the junction of four major phytogeographic units [35], this region shows a high level of habitat heterogeneity in a small area (440 km^2^) with a mosaic of forest types ranging from semi-arid scrub to evergreen rain forest. The forest is highly fragmented and surrounded by a complex matrix of crops, meadows, zebu cattle grazing pastures and a network of riparian corridors [36], [37]. We collected faecal pellets immediately after defecation from 96 individuals belonging to 63 family groups (1 to 4 individuals per group) in the twelve main forest complexes within the golden-crowned sifaka’s distribution (**Fig. 1**). Sampling was carried out during two field missions in June-July-August 2006 and 2008, both during the dry season. All details about sample collection are provided in [38]. *P. tattersalli* has a relatively long transit time (20 ± 4.8 hr) with a retention time (average amount of time a chemical marker was retained in the gut) of more than 36 hours [39]. Therefore, we can expect that the DNA present in the faecal pellets is a good proxy for the plant ingested in the few days preceding the sampling.

### Habitat characterization

Landscape structure was described using a GIS, with a 30×30 m grid cell size, constructed using a Landsat images taken in 2002 (April 15 and May 27). We used the Normalized Difference Vegetation Index (NDVI), calculated as follow: (NIR-Red)/(NIR+Red) (where NIR is TM4) to remove shade effects, the area of interest being in a hilly landscape. The vegetation map (performed by CC, LG, LN and PR) was obtained by automatic classification of five channels of LANDSAT with 56 classes using the module ISOCLUSTER from the IDRISI computer program (Clark Labs). The classes corresponding to the same types of vegetation were then regrouped to obtain 30 classes. Field knowledge and observations, aerial images from 1949 and 1992 and very high resolution data for part of the area (SPOT imagery, QuickBird Imagery from Google Earth) were used to control the reliability of the classification and improve its accuracy. We distinguished seven main habitat types (see **Table 1**): dry deciduous forest, mesophyllous forest, ombrophilous forest, sclerophyllous forest, ripicolous forest (in decreasing order of surface importance among the whole area) as well as secondary forest of each of these formations and matrix habitats (*i.e.* savannas, meadows, pastures, agricultural fields, shrubs, settlements, bare soil). To estimate the habitat composition within the group’s home range, we calculated the proportion of surface covered by each of these habitats within a circular buffer of 500*m* of radius centered on the sampling location. We thus considered home ranges of size 20 ha in agreement with the range of values observed by Meyer [34] although we are conscious that this may vary widely depending on habitat alteration, population density and resource availability [17]. In the following, we considered the proportion of matrix habitats as the level of openness of the habitat.

**Table 1:** Relative proportion of the most important habitat types within *P. tattersalli* home ranges.

Habitat type	Description	Mean proportion (sd)	N_maj_
Dry deciduous forest	**Situation**: slopes	22 % (3.5)	17
	**Altitudinal range**: mean: 440(+/- 206) m, 40– 1073 m.		
	**Forest height**: 10-15 m, emergent trees up to 19 m.		
	**Seasonal variation**: deciduous leaves, some sclerophyllous species.		
	**Particularities**: many adaptations to drought (accumulation of water).		
	**Representative species**: *Ambilobea madagascariensis*, *Xyloolaena perrieri*		
Mesophylous forest	**Situation**: mainly slopes	13% (2.9)	8
	**Altitudinal range**: mean: 225 (+/- 88) m, 30– 600 m		
	**Forest height**: 13–17 m, emergent trees up to 22 m		
	**Seasonal variation**: deciduous and persistent leaves		
	**Particularities**: a very large species diversity occurs in this formation		
	**Representative species**: *Euphorbia madravioky* and *Celtis mildbraedii*		
Sclerophyllous forest	**Situation**: plain	8 % (2.2)	5
	**Altitudinal range**: mean: 50 (+/- 40) m, 20– 160 m		
	**Forest height**: 7–9 m, emergent trees up to 12 m		
	**Seasonal variation**: deciduous and persistent leaves		
	**Particularities**: many species with coriaceous and spiny leaves		
	**Dominant** **species**: *Diospyros danguyana, Stachyandra viticifolia*		
Ripicolous forest	**Situation**: stream beds in plains and slopes	17 % (1.9)	8
	**Altitudinal** **range**: mean: 130 (+/- 60) m, 20– 620 m		
	**Forest** **height**: 13–15 m, emergent trees up to 30 m		
	**Seasonal variation**: leaves mainly persistent with some deciduous trees		
	**Particularities**: among the largest trees of the Daraina region		
	**Representative species**: *Syzygium sakalavarum*, *Canarium madagascariense*		
Secondary forest	**Situation** : plains and slopes	2 %(0.8)	1
	**Altitudinal range: mean: 450 (+/- 280) m, 20**– **1020 m**		
	**Forest height**: 5–15 m, emergent trees up to 8–20 m Seasonal variation: deciduous and persistent leaves		
	**Particularities**: vegetation and species related to the respective original forest type		
Ombrophilous forest	**Situation**: mountain ridges (a) and upperslopes (b)	0%	0
	**Altitudinal range**: 750–930 (+/- 180) m, 70– 1171 m		
	**Forest height**: 4–10 m (a), 20–25 m (b), emergent trees up to 30 m		
	**Seasonal variation**: persistent leaves.		
	**Particularities**: important cloudiness during the whole year, some rare observations of the *Propithecus tattersalli*		
	**Representative species**: *Chrysophyllum boivinianum* and *Dypsis nodifera*		
Matrix habitats	**Situation**: mainly plains	38 % (2.7)	24
	**Altitudinal range**: 15–400 m		
	**Forest height**: 0.3–3 m, isolated emergent trees up to 10 m		
	**Seasonal variation**: mainly deciduous		
	**Representative species**: *Poaceae* ssp., *Mascarenhasia arborescens*		

Proportions were calculated within circular buffers of 500m of radius centered on the group sampling location. N_maj_ indicate the number of groups for which this habitat is the most represented.

### Laboratory procedures

The diet analysis was carried out using the *trn*L approach [30]. DNA extraction was performed using ca. 50 mg from the outer layer of dried faecal pellets in a Class II microbiological hood and following the 2CTAB/PCI protocol adapted from Vallet et al. [40]. Mock extractions without samples were systematically performed to monitor possible contaminations. DNA amplifications were carried out in a final volume of 50 µL, using 4 µL of DNA extract diluted 100 times as template. The amplification mixture contained 1 U of AmpliTaq® Gold DNA Polymerase (Applied Biosystems, Foster City, CA), 10 mM Tris-HCl, 50 mM KCl, 2 mM of MgCl2, 0.2 mM of each dNTP, 0.1 µM of each primer, and 0.005 mg of bovine serum albumin (BSA, Roche Diagnostic, Basel, Switzerland). The mixture was denatured at 95°C for 10 min, followed by 45 cycles of 30 s at 95°C, and 30 s at 55°C; as the target sequences are usually shorter than 100 bp, the elongation step was removed. We amplified the P6 loop region of the *trn*L (UAA) intron [41] using the universal primers g (5′-GGGCAATCCTGAGCCAA-3′) and h (5′-CCATTGAGTCTCTGCACCTATC-3′) modified by the addition of specific tags on the 5′ end to allow the assignment of sequence reads to the relevant sample [30]. All the PCR products were tagged identically on both ends. These tags were composed of CC on the 5' end followed by nine variable nucleotides that were specific to each sample. The nine variable nucleotides were designed using the oligoTag program (http://www.prabi.grenoble.fr/trac/OBITools) with at least three differences among the tags, without homopolymers longer than two, and avoiding a C on the 5' end. All the PCR products from the different samples were first titrated using capillary electrophoresis (QIAxel, QIAgen GmbH, Hilden, Germany) and then mixed together, in equimolar concentration, before the sequencing. The sequencing was carried out on the Illumina/Solexa Genome Analyzer IIx (Illumina, San Diego, California), using the Paired-End Cluster Generation Kit V4 and the Sequencing Kit V4 (Illumina, San Diego, California), and following manufacturer's instructions. A total of 108 nucleotides were sequenced on each extremity of the DNA fragments.

### Completion of the DNA barcoding taxonomic reference library

We built a local DNA barcoding reference library of genera by retrieving the whole *trn*L (UAA) intron sequence from EMBL for the plant genera present in the Daraina region, using a checklist of 2039 species belonging to 654 genera and 146 families compiled by the Botanical Garden of Geneva (performed by CC, LG, LN and PR). We decided to build a local database at the genus level and not at the species level for three main reasons. First, the P6 loop region of the *trn*L has a relatively small size which makes it particularly suitable for analyzing diet from degraded DNA [29] but this leads to a relatively low resolution at the species level [41], [42]. Secondly, a large number of species of the checklist are endemic of Madagascar and their sequences of *trn*L (UAA) intron are often not available in EMBL. Lastly, due to the complexity of the plant taxonomy in Madagascar, numerous plants of the checklist have not been identified to the species level. As a “consensus sequence” for each genus, we tried, as much as possible, to retrieve a sequence of one of the species of this genus present in the Daraina region (28% of the genera of the area). In the cases where it was not possible, we retrieved a sequence of another species of the same genus present in Madagascar (15% of the genera). If no sequence of Malagasy species was available in EMBL, we retrieved a sequence of the same genus regardless of its geographical origin (26% of the cases). In addition, we collected 224 plants from 40 plots distributed across the entire Daraina region, which were identified by botanists of the Herbarium of Antananarivo and the Botanical Garden of Geneva. These samples correspond to 114 genera (of which 58% were identified to the species level) belonging to 36 different families. For each sample, a piece of leaf was preserved in silicagel gel before DNA extraction. Total DNA was extracted with the BioSprint DNA Plant Kit (Qiagen Gmbh, Hilden, Germany) following the manufacturer’s instructions. DNA extracts were then amplified with universal c-d primer pair [43] amplifying the whole *trn*L (UAA) intron (c: 5'-CGAAATCGGTAGACGCTACG-3'; d: 5'-GGGGATAGAGGGACTTGAAC-3'). The PCR products obtained were sequenced on an ABI PRISM_ 3100 Genetic Analyzer (Applied Biosystems, Foster City, CA) using standard protocols [41]. All the sequences have been deposited in GenBank (accession nos. KC479210 - KC479323). Finally, for each reference plant, the P6 loop sequences (21–132 bp) were extracted from the whole *trn*L (UAA) intron sequence and used to build the reference database. Overall, the local database contained 449 genera (69% of the genera of the checklist) belonging to 112 families. Approximatively 23% of the families on the checklist are not represented in the local database, of which most endemic to Madagascar, monogeneric or bigeneric (29 out 34). The overall discrimination rate of the database at the genus level (R_g_  =  total number of unique sequences/total number of genera in the database) was 0.83. This means that this local database can identify without ambiguity 57% (i.e 0.83*0.69) of the genera present in the study area.

### Sequence analysis and taxon assignation

The sequence reads from faecal DNA were analyzed using OBITools and associated programs (www.prabi.grenoble.fr/trac/OBITools). First, the direct and reverse reads corresponding to a single molecule were aligned and merged using the solexaPairEnd program, taking into account quality data during the alignment and the consensus computation. Then, primers and tags were identified using the ngsfilter program. Only sequences with perfect match on tags and a maximum of two errors on primers were taken into account. The amplified regions, excluding primers and tags, were kept for further analysis. Strictly identical sequences were clustered together using the obiuniq program, keeping the information about their distribution among samples. Sequences shorter than 10 bp, or containing nucleotides other than A, C, G and T, or with occurrence lower or equal to 10 were excluded using the obigrep program. To eliminate the sequences potentially resulting from PCR artefacts, we applied two additional cleaning steps: First, we discarded all the sequences with a total number of counts less than 1% of the most common sequence and the sequences for which the number of counts *per* sample was always lower than 10. Second, we assigned a status to the different sequences in each PCR (or sample) using the following rule: “H” (header) means that there is no other variant (with a single difference) with a higher count of this sequence in the PCR, “S” (single) means that there is no other variant with a single difference) in the PCR, “I” (internal) means that another variant (with a single difference) with higher count is present in the PCR. All the sequences with “S” or “H” in more than 95% of the samples containing the relevant sequence (criterion 1) and 95% of the total number of counts (criterion 2), were automatically retained. The sequences that meet only one of these two criteria were classified as ambiguous and were manually checked. The remaining sequences were discarded from the dataset. To assign a taxon to each of the filtered sequences, we successively used two different taxonomic reference libraries. The first database (“EMBL database”) was built by extracting all the sequences of P6 loop of the *trn*L intron from EMBL nucleotide library using the ecoPCR program [44]. The second database (“local database” - see above) included only the sequences of the plant found in the Daraina region. In both cases, the taxon assignation was achieved using the EcoTag program [28]. EcoTag relies on Needleman and Wunsch [45]’s dynamic programming global alignment algorithm to find highly similar sequences in the reference database. A unique taxon was assigned to each unique sequence. This unique taxon corresponds to the last common ancestor node in the NCBI taxonomic tree of all the taxids that best matched against the query sequence. A genus name was accepted only if the best match score was equal or greater to 0.98 or more and a family name was retained only if the maximum identity was equal or above 0.95 or more. We did not consider the sequences with a maximum identity < 0.9 which were likely the results of PCR artefacts (including chimeras, primer dimers or nuclear pseudogenes) or contaminations. Lastly, the list of taxa identified using the local reference database was evaluated by one of us (LN) who is a taxonomist specialist of the Daraina flora. This allowed us, in some cases, to refine, for example, the identification to a single genus when there was an ambiguity among two genera with the same DNA barcode but only one genus was likely to occur in the sampling site and/or to be consumed by sifakas.

### Determinants of diet richness and composition across landscape

To evaluate the efficiency of our sampling effort, we analysed the cumulated number of both unique sequences and genera in the faeces against the number of individuals sampled using accumulation models [46]. Rarefaction curves were generated using 500 “Mao Tau” randomizations with EstimateS Version 8.2 [47]. Total richness was estimated by the Chao2 estimator [48]. We then used a multiple linear regression to model the variation of the sequence richness across the different habitats, using the proportions of the six habitat types within the home range centered on the location of the sample collection. Lastly, to measure the relationship between the diet composition and the habitat composition, we employed a coinertia analysis [49] (as implemented in the ade4 R package software [50]) between the PCA of the diet composition table (presence/absence of each food item in each sample) and the PCA of the landscape structure table. In the co-inertia analysis, the diet and landscape tables are represented in a common space in order to maximize their covariance. Then, we tested the significance of the co-inertia using a Monte-Carlo permutation test (N = 1000).

## Results

**Figure 2:**
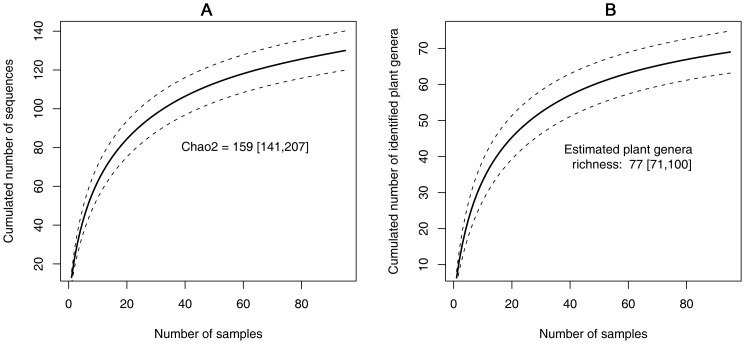
Plant richness rarefaction curve. The solid line represents the cumulated number of MOTUs (a) and food plant genera (b) as a function of the number of sampled individuals. The dashed lines are the 95% confidence intervals.

The next-generation sequencing produced 2,260,389 reads (mean =  23,793 reads per faecal sample, SD  =  1192, range = 2442-51402) corresponding to 4604 unique sequences. After applying the different filtering steps, we finally retained 130 unique sequences (or MOTUs for Molecular Operational Taxonomic Units) with a total count of 927,706 reads (41% of the initial total count) **(Table S1)**. We did not observe any relation between the number of reads per sample and the unique sequence richness (Pearson’s r =  0.13, p  =  0.19 > 0.05). Each faecal sample contained an average of 13.9 unique sequences (±3.8 sd). The cumulative numbers of MOTUs reached an asymptote (Chao2  =  159 [141 – 207](sd)) suggesting that increased sampling would not bring major changes in the information about the diet richness and composition of golden-crowned sifakas **(** **Fig. 2** **)**.

### Diet richness and composition of the golden-crowed sifakas

**Figure 3:**
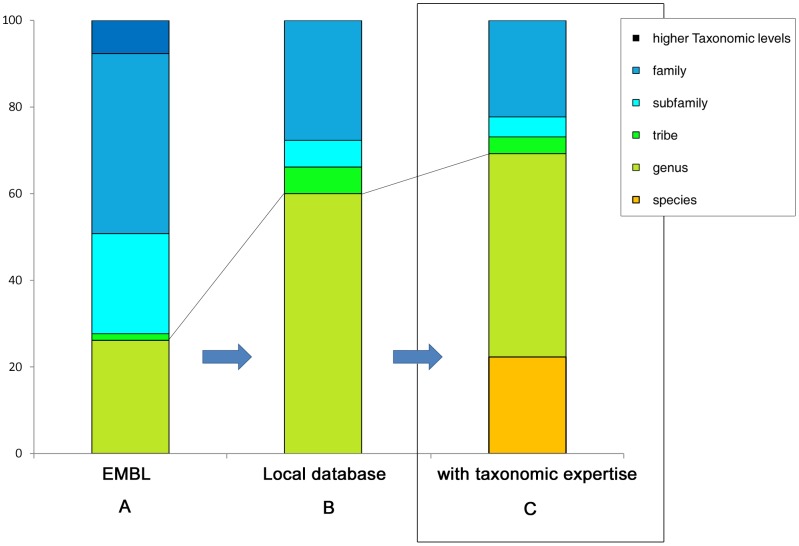
Taxonomic resolution of the golden-crowned sifaka diet. Panel (a) presents the results base on the EMBL reference database only. Panel (b) presents the results using both EMBL and customized local reference database. Panel (c) uses the final results integrating the final validation by taxonomic experts. The proportions correspond to the number of sequences assigned to each taxonomic rank compared to the total number of identified sequences from DNA Barcoding (N = 130).

The diet of *P. tattersalli* included at least 130 different plants (**Table S1**) belonging to 80 genera and 49 different families (**Table 2**) and showed a high flexibility with an average frequency of occurrences (F_o_, the proportion of faeces including the MOTU) of only 9.8 % (sd =  0.12). This means that most plants were only found in a limited number of the individuals. Eighty plants (61% of the MOTUs) were identified to the genus level (**Fig. 3c**) among which 29 are monospecific in the Daraina region (*i.e.* we could unambiguously assign a species name to them). Five MOTUs were identified at best at the subfamily level and 29 at the family level. The 17 remaining MOTUs (13%) were assigned to a higher taxonomic level. Importantly we found that by using both the local reference database and the expertise of a taxonomist of the Daraina flora, we improved the accuracy of the identification for 52% of the sequences and we more than doubled the number of MOTUs identified to the genus level (from 29 to 80) (see and compare **Fig. 3** **a,b,c**). The most consumed plant/sequence belonged to the family of *Apocynaceae* (with a frequency of occurrence F_o_ of 80% and a global frequency F_s_ of 30%). However, we could not assign a genus name to the corresponding sequence because of the weak power of resolution of the P6 loop within the *Apocynaceae* (the discrimination rate (R_g_) of this family in the reference database is 0.69). Indeed, this sequence matched perfectly with the P6-loop barcodes of five different genera and may correspond to either one or several of them. A similar pattern was observed in the *Sapindaceae* (R_g_  =  0.62) for which only two sequences (among the five assigned to this family) could be identified at the genus level. Overall, six MOTUs could not be assigned to the genus level because of an ambiguity between two genera showing exactly the same P6-loop barcode. Within the overall 10 top food plants, we identified the genera *Poupartia*, *Olax*, *Landolphia*, *Marsdenia* and *Dalbergia* (**Table S1**). Interestingly, 14 of the 80 identified genera contain introduced or cultivated plants among which we can cite the mango (*Mangifera indica*) (18 occurences), the banana (*Musa* sp. 13 occ.) or plants of the genera *Vigna* (27 occ.), *Solanum* (13 occ.) or *Anacardium* (9 occ.).

**Table 2:** List of food-plant families.

Rank	Family Name	Nb MOTUS	Nb Ind	F_o_	Nb Count	F_s_
1	*Fabaceae*	33	91	0.96	283131	0.15
2	*Apocynaceae*	8	89	0.94	809106	0.44
3	*Anacardiaceae*	7	47	0.49	163491	0.09
4	*Olacaceae*	2	50	0.53	85898	0.05
5	*Sapotaceae*	1	46	0.48	29609	0.02
6	*Sapindaceae*	5	40	0.42	112168	0.06
7	*Ebenaceae*	2	30	0.32	25919	0.01
8	*Poaceae*	5	26	0.27	5475	0
9	*Primulaceae*	3	25	0.26	25617	0.01
10	*Convolvulaceae*	3	23	0.24	6477	0
11	*Moraceae*	4	21	0.22	59990	0.03
12	*Meliaceae*	1	20	0.21	6593	0
13	*Burseraceae*	2	19	0.2	6719	0
14	*Solanaceae*	2	17	0.18	12056	0.01
15	*Melastomataceae*	1	16	0.17	6280	0
16	*Brassicaceae*	2	15	0.16	3642	0
17	*Combretaceae*	1	15	0.16	1082	0
18	*Cucurbitaceae*	2	15	0.16	11773	0.01
19	*Physenaceae*	1	15	0.16	15726	0.01
20	*Erythroxylaceae*	1	14	0.15	2101	0
21	*Araliacae*	1	14	0.15	13346	0.01
22	*Musaceae*	1	13	0.14	24795	0.01
23	*Rubiaceae*	2	13	0.14	22961	0.01
24	*Rutaceae*	3	13	0.14	3083	0
25	*Annonaceae*	3	11	0.12	1782	0
26	*Phyllanthaceae*	1	11	0.12	45568	0.02
27	*Loranthaceae*	2	8	0.08	549	0
28	*Asteraceae*	5	7	0.07	1405	0
29	*Celastraceae*	2	7	0.07	816	0
30	*Myrtaceae*	2	6	0.06	2093	0
31	*Oleaceae*	2	6	0.06	1174	0
32	*Rhamnaceae*	2	6	0.06	14527	0.01
33	*Sphaerosepalaceae*	1	6	0.06	737	0
34	*Lauraceae*	1	5	0.05	954	0
35	*Plantaginaceae*	1	5	0.05	4437	0
36	*Lecythidaceae*	2	4	0.04	370	0
37	*Malvaceae*	1	4	0.04	3219	0
38	*Apiaceae*	1	3	0.03	425	0
39	*Asparagaceae*	1	3	0.03	250	0
40	*Pittosporaceae*	1	3	0.03	9409	0.01
41	*Putranjivaceae*	1	3	0.03	935	0
42	*Euphorbiaceae*	1	2	0.02	144	0
43	*Lamiaceae*	1	2	0.02	7771	0
44	*Montiniaceae*	1	2	0.02	432	0
45	*Araceae*	1	1	0.01	117	0
46	*Capparaceae*	1	1	0.01	124	0
47	*Connaraceae*	1	1	0.01	430	0
48	*Pedaliaceae*	1	1	0.01	229	0
49	*Salicaceae*	1	1	0.01	1214	0

Families were ranked by their frequency of occurrences (F_o_), F_s_ values correspond to the frequencies of the whole sequences assigned to these families.

### Spatial variability in diet diversity and composition

**Figure 4:**
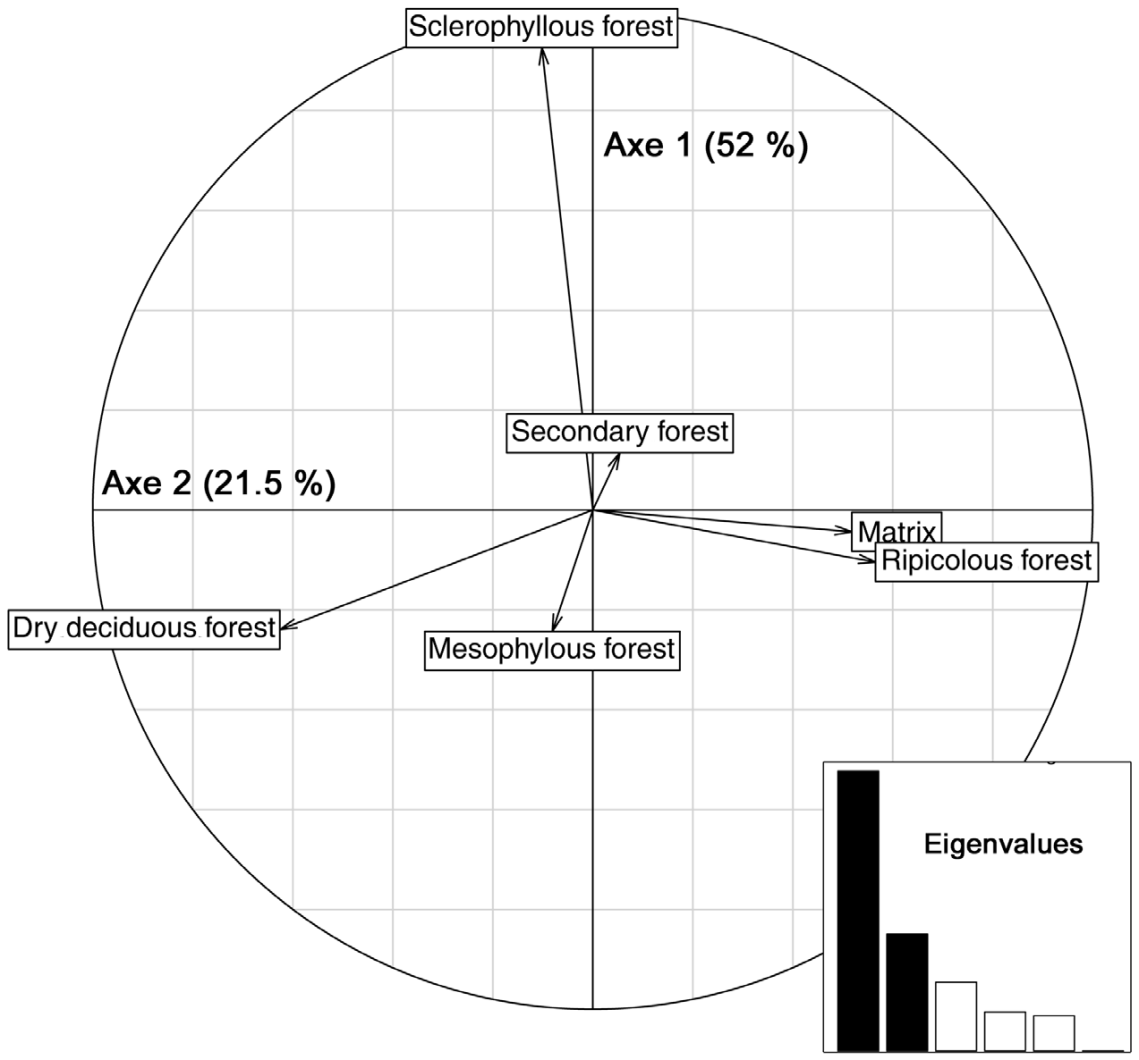
Environmental determinants of diet composition. Biplots of the co-inertia analysis between the PCA of diet composition (presence/absence of the 130 taxa) and the PCA of landscape structure (% area of the six habitats within the circular buffers).

The sequence richness per individual was not spatially autocorrelated (Moran test, p  =  0.136 > 0.05) and was weakly influenced by landscape structure (Linear regression R^2^  =  0.066 - F-statistic: 2.058 on 6 and 88 DF, p-value: 0.066). Individuals of a same group have a very similar diet (i.e. only 1.7% of the total inertia is due to the within group PCA). Most of the dietary differences (98.3% of the total inertia) are observed among groups in relation with habitat type and openness. Indeed, the co-inertia between landscape structure and diet composition was moderate (RV  =  28.9%) and significant (P = 0.001 - Monte-Carlo test Based on 999 replicates) (**Fig. 4**). The two main axes represented 74% of the total inertia. The main axis (52.5% of the inertia) opposed edge areas (secondary forests, matrix habitats, riparian forests), which co-occurred with the genus *Poupartia*, Mango and cashew trees with more wooded areas (mesophylous or dry deciduous forests). The second axis (21.5%) opposed the sclerophyllous forests found in the North-East of the Daraina region from other habitats and correlated positively with the genera *Ardisia*, *Cedrelopsis*, *Ipomoea* and some unidentified plants of the Rubiaceae family (MOTU 45). The canonical scores of the different plants for the three first axes are in Table S2.

## Discussion

### A highly diverse diet

In this study, we used an innovative approach combining DNA barcoding and Illumina next-generation sequencing to examine the diet diversity and plasticity of a rare and critically endangered lemur primate across its entire fragmented range. Our results suggest that golden-crowned sifakas consume a wide range of plants selected in various habitats both on the edge and centre of the fragments. In the dry season, which is the only season for which we had data, the species was found to feed on at least 130 food plants belonging to 80 genera and 49 different families. It has to be noted that this estimate is very conservative. Indeed, we used a computational approach with successive filtering steps in order to eliminate all sources of erroneous reads (PCR-generated chimeric sequence, primer dimmers, nuclear pseudogenes or contaminations - see also [29] for a review). This led to the elimination of 97% of the unique sequences.

Our molecular assessment of diet diversity appears consistent with the number obtained by Meyers (153 food species in 88 genera) [34] who used direct observations to analyze the feeding behavior of six family groups of *P. tattersalli* inhabiting three forest fragments in the South-East of the Daraina region. However, when we compare the composition of the two lists, we observed both significant overlap and differences. We identified 44% of the genera found by Meyers (N = 32) and 64% (N = 50) of the genera if we consider all the taxa perfectly matching with the P6-loop sequences in the faeces (i.e. including the ambiguous barcodes matching more than one genus). Furthermore, when we limited the comparison of our list with the 10 top key-food plants (in term of feeding time) in each of the sites studied by Meyers, the proportion of common genera increased from 44 to 58% and 79% if we take into account the ambiguous barcodes (**Table 3**). Our list contained also 47 new genera (**Table S1**) among which 11 (23%) are cultivated or naturalized plants. We are conscious that this comparison has to be taken with caution since the spatial and temporal frameworks of the two studies differ strongly. Indeed, Meyers conducted his study in both dry and humid seasons (150 accumulated days of direct observations across a year). Some plants consumed for their flowers and fruits during the wet season are probably not eaten for their leaves during the dry season and thus could not be found in the faeces during our sampling period. Furthermore, Meyers analyzed the diet of only six family groups in three forest fragments and therefore could not capture all the variability of the resource availability at the landscape scale.

**Table 3:** List of genera found in common with the study of Meyers [34].

Genus	Nspecies	Mean Rank	F_o_
*Acacia*	1	22	0.14
*Albizia*	4	11	0.04
*Bakerella*	2	18	0.08
*Baudouinia*	1	1	0.02
*Dupuya*	2	8	0.09
*Cynometra*	3	8	0.17
*Dichrostachys*	1	6	0.04
*Diospyros*	6	12	0.27
*Drypetes*	1	24	0.03
*Ficus*	1	12	0.08
*Filicium*	1	1	0.14
*Foetidia*	1	21	0.03
*Grewia*	2	9	0.04
*Landolphia*	1	19	0.37
*Mangifera*	1	8	0.19
*Olax*	2	10	0.52
*Poupartia*	2	17	0.56
*Protorhus*	2	19	0.14
*Schefflera*	3	20	0.15
*Terminalia*	4	18	0.16
*Xanthocercis*	2	13	0.13

We considered as the top food-plants in Meyers study the genera that accounted for at least 1% of the feeding time of the studied groups. For each genus, we indicated the number of corresponding species in Meyers’ list and their mean rank in term of time spent feeding on these plants. F_o_ is the frequency of occurrence of these genera in our study (i.e. proportion of individuals consuming the genus).

### A high level of dietary plasticity

Overall, our findings are in agreement with our initial expectation that *P. tattersalli* has a very plastic response to landscape heterogeneity and thus confirm our previous results on species dispersal [32] and space use [33]. Indeed, we did not detect an influence of landscape structure on diet richness suggesting that individuals are able to exploit resources in both matrix and forest habitats as illustrated by the presence of numerous non-indigenous plants in their diet. Furthermore, we observed large shifts in dietary composition in relation with habitat composition and openness, hence suggesting a high flexibility of foraging strategies. These results support the prediction that predominantly folivorous primates are more edge-tolerant than strictly frugivorous species as suggested by previous studies on howler monkeys [51], red colobus [52] or black and white colobus [53]. Indeed, fruits may become scarce due to logging or changes in micro-climates associated with edge effects [8], [54]. The ability of primates to maintain viable populations in forest fragments appear also to be related to the composition of the matrix habitats, the human population density and hunting practices [15], [55]. For instance, the high human population densities in the matrix habitats in the region of Kibale (Uganda) deters grey-cheeked mangabeys *(Cercocebus albigena*) from utilizing the matrix resources and the species is absent from all the forest fragments outside the national park. Similar results were observed in Chimpanzees whose individuals inhabiting the small fragments outside the Budongo forest (Uganda) are often killed by farmers if they are caught raiding agricultural fields [56]. In our case, the human density is very low and the species is protected by a fady (traditional taboo) preventing its hunting by local populations [57]. Lastly, we noted that Meyers ‘study [34] identified a large seasonal shift in *P. tattersalli* foraging strategies in term of parts exploited for food (e.g. young or mature leaves, fruits or seeds). This emphasizes the interest of combining DNA-based approach with a more conventional micro-histological study to obtain a more comprehensive picture of the dietary plasticity.

### Power and limitations of the DNA metabarcoding approach for the diet analysis of tropical mammals

The DNA metabarcoding approach used here presents several advantages in comparison with traditional approaches such as direct observation of foraging behavior or microscopic examination of gut content or faeces. First, it is particularly time and cost effective and allowed to assess the dietary profile of a large number of individuals simultaneously. This provides the opportunity to conduct large scales analyses hence making easier the study and understanding of the processes shaping the pattern of diet diversity at the landscape scale. DNA-based approaches are also renowned because they do not require strong taxonomic skills to identify the food taxa [29]. However, we showed here that the expertise of taxonomists is particularly valuable to control the reliability of the results and improve the accuracy of the taxonomic identification [58]. In our case, the presence of a detailed checklist of the plants of the study area considerably reduces the range of putative food plants by building a customized local reference. This allowed us to resolve a larger number of ambiguities and double the numbers of MOTUs identified at the genus or species level. Improving the level of completeness of reference databases is probably the most critical issue in DNA-based diet analyses. We are conscious that building exhaustive species databases is probably out of reach in tropical forests [59] due to the often high diversity of the flora and the hard and challenging taxonomic work (less than two-thirds of the plants collected for this study could be identified at the species level). However, we think that particular attention should be paid to obtaining representative databases at least at the family level to limit misidentifications or unrecognized sequences. For instance, none of the *trnL* sequence of the family *Physenaceae* (endemic of Madagascar) was available in the public databases. Consequently, when we assigned the taxon names using the EMBL database only, we removed by mistake a DNA sequence of this plant family because its maximum identity (0.81) was below our exclusion threshold (i.e. <0.9). There are also cases where the public database may help in identifying the sequences that would not be assigned (and hence discarded) by using the reference databases. This happens when the plants are not known to exist in the study area but actually do [42]. In our study, the public database enables the identification of numerous cultivated or introduced plants that were not reported in the checklist of the taxonomists. A limitation of this approach is the accuracy of the identification of the P6-loop barcode which was found to differ strongly between plant families and does not always allow us to reach the genus level. This marker presents numerous advantages: it has a small length and can be easily amplified despite the high DNA degradation in faecal samples and a large taxonomic coverage (i.e. primers can amplify a wide range of plants). However, its resolution capacity was found to be particularly low within some major families such as *Apocynaceae* or *Sapindaceae* with the risk of underestimating the diet richness of this species. Consequently, some efforts must be made in future to increase the resolution power of the method may be by using additional DNA barcodes with a more restricted taxonomic coverage (i.e. using primers specific to these families) but with a higher resolution within these families (i.e. hierarchical barcoding – see [29]). Lastly, although this is unlikely to have extensively biased the results, some environmental contaminations from the forest floor or debris blown is always possible despite our precautions to collect fresh pellets immediately after defecation and to clean their outer layer before the DNA extraction.

## Conclusion and Perspectives

This study illustrated that DNA-metabarcoding approach is a very powerful tool to rapidly gather data on the species feeding resources. This can have many implications. For instance a comparison with data on human plant use (for food, medicine, building materials, and coal) could help in identifying putative conflicts of interest and guide management strategies. This non-invasive approach is particularly relevant for studying the diet of elusive species that correspond to the large majority of endangered primate species for which observational data are nearly impossible to obtain. Overall, our results suggest that golden-crowned sifakas have a generalist and plastic diet that help it to survive in a human-dominated mosaic landscape. However, we are conscious that the results obtained here have to be treated with caution. Indeed, the recent political crisis in Madagascar has led to the increase of poaching, mining, selective logging and habitat alteration in the Daraina region. Furthermore, we have no data on the minimum requirements of the species and on the availability of the plants in the different environments. Without a comparative study of the viability and breeding rates of individuals inhabiting edge and core areas, it is hard to definitely conclude on the long-term ability to cope with ongoing environmental changes.

## Supporting Information

Table S1 **List of the lowest taxonomic levels assigned to the 130 MOTUs found in the** ***P. tattersalli*** **faecal pellets**. MOTUs were ranked by their frequencies of occurrences (F_o_). F_s_ are the frequency of sequences. New taxa are indicated in bold type.
(DOCX)

Table S2 **Canonical scores of the different MOTUs for the two first axes of the co-inertia analysis.**
(DOCX)
