# Supplementary material for: A DNA Metabarcoding Study of a Primate Dietary Diversity and Plasticity across Its Entire Fragmented Range
Source: PLoS One. 2013 Mar 19;8(3):e58971. doi: 10.1371/journal.pone.0058971 (PMC3602585; doi:10.1371/journal.pone.0058971)
Supplement: Table S1 — List of the lowest taxonomic levels assigned to the 130 MOTUs found in the P. tattersalli faecal pellets. MOTUs were ranked by their frequencies of occurrences (Fo). Fs are the frequency of sequences. New taxa are indicated in bold type. (DOCX) [file pone.0058971.s001.docx]

**Table S1. List of the lowest taxonomic levels assigned to the 130 MOTUs found in the *P. tattersalli* faecal pellets**. MOTUs were ranked by their frequencies of occurrences (F_o_). F_s_ are the frequency of sequences. New taxa are indicated in bold type.

| **MOTU ID** | **Lowest taxonomic levels** | **Taxon Type** | **Numb of Ind.** | **F_o_ (%)** | **Nb of Counts** | **F_s_ (%)** |
| --- | --- | --- | --- | --- | --- | --- |
| **1** | *Apocynaceae* | Family | 76 | 0.8 | 549372 | 0.3 |
| **2** | *Poupartia* | Genus | 53 | 0.56 | 89818 | 0.05 |
| **3** | *Olax* | Genus | 49 | 0.52 | 84575 | 0.05 |
| **4** | *Sapotaceae* | Family | 46 | 0.48 | 29609 | 0.02 |
| **5** | ***Marsdenia*** | Genus | 37 | 0.39 | 46906 | 0.03 |
| **6** | *Landolphia* | Genus | 35 | 0.37 | 20452 | 0.01 |
| **7** | *Apocynaceae* | Family | 34 | 0.36 | 76597 | 0.04 |
| **8** | *Mimosoideae* | Subfamily | 31 | 0.33 | 26667 | 0.01 |
| **9** | *Fabaceae* | Family | 29 | 0.31 | 5594 | <0.01 |
| **10** | *Dalbergia* | **Genus** | 27 | 0.28 | 18678 | 0.01 |
| **11** | ***Vigna unguiculata*** | Species | 27 | 0.28 | 14932 | 0.01 |
| **12** | *Diospyros* | Genus | 26 | 0.27 | 19435 | 0.01 |
| **13** | *Sapindaceae* | Family | 24 | 0.25 | 41916 | 0.02 |
| **14** | ***Ardisia capuronii*** | Species | 23 | 0.24 | 25339 | 0.01 |
| **15** | *Caesalpinieae* | Tribe | 21 | 0.22 | 22847 | 0.01 |
| **16** | *Triticeae* | Tribe | 21 | 0.22 | 3082 | <0.01 |
| **17** | *Meliaceae* | Family | 20 | 0.21 | 6593 | <0.01 |
| **18** | *Anacardiaceae* | Family | 19 | 0.2 | 652 | <0.01 |
| **19** | *Mangifera indica* | Species | 18 | 0.19 | 40115 | 0.02 |
| **20** | *Mimosoideae* | Subfamily | 18 | 0.19 | 7846 | <0.01 |
| **21** | ***Gagnebina*** | Genus | 17 | 0.18 | 5077 | <0.01 |
| **22** | *Cynometra* | Genus | 16 | 0.17 | 76819 | 0.04 |
| **23** | *Melastomataceae* | Family | 16 | 0.17 | 6280 | <0.01 |
| **24** | *Physena* | Genus | 15 | 0.16 | 15726 | 0.01 |
| **25** | *Terminalia* | Genus | 15 | 0.16 | 1082 | <0.01 |
| **26** | *Schefflera* | Genus | 14 | 0.15 | 13346 | 0.01 |
| **27** | *Burseraceae* | Family | 14 | 0.15 | 4292 | <0.01 |
| **28** | ***Crateva greveana*** | Species | 14 | 0.15 | 3507 | <0.01 |
| **29** | *Erythroxylum* | Genus | 14 | 0.15 | 2101 | <0.01 |
| **30** | *Filicium longifolium* | Species | 13 | 0.14 | 69346 | 0.04 |
| **31** | ***Musa*** | Genus | 13 | 0.14 | 24795 | 0.01 |
| **32** | ***Abrahamia*** | Genus | 13 | 0.14 | 19724 | 0.01 |
| **33** | ***Solanum*** | Genus | 13 | 0.14 | 11537 | 0.01 |
| **34** | *Cucurbitaceae* | Family | 13 | 0.14 | 7451 | <0.01 |
| **35** | *Acacia* | Genus | 13 | 0.14 | 6711 | <0.01 |
| **36** | *Xanthocercis madagascariensis* | Species | 12 | 0.13 | 15714 | 0.01 |
| **37** | ***Trilepisium madagascariense*** | Species | 12 | 0.13 | 6415 | <0.01 |
| **38** | ***Ipomoea*** | Genus | 12 | 0.13 | 2320 | <0.01 |
| **39** | ***Bridelia pervilleana*** | Species | 11 | 0.12 | 45568 | 0.02 |
| **40** | *Neoapaloxylon* | Species | 11 | 0.12 | 41810 | 0.02 |
| **41** | *Tabernaemontana* | Genus | 11 | 0.12 | 2339 | <0.01 |
| **42** | *Anacardium occidentale* | Species | 9 | 0.09 | 12152 | 0.01 |
| **43** | *Dupuya madagascarensis* | Species | 9 | 0.09 | 3714 | <0.01 |
| **44** | *Apocynaceae* | Family | 8 | 0.08 | 96257 | 0.05 |
| **45** | *Rubiaceae* | Family | 8 | 0.08 | 21170 | 0.01 |
| **46** | *Ficus* | Genus | 8 | 0.08 | 20701 | 0.01 |
| **47** | *Fabaceae* | Family | 7 | 0.07 | 10120 | 0.01 |
| **48** | *Merremia* | Genus | 7 | 0.07 | 3510 | 0 |
| **49** | ***Entada*** | Genus | 7 | 0.07 | 486 | <0.01 |
| **50** | *Cedrelopsis* | Genus | 6 | 0.06 | 1637 | <0.01 |
| **51** | *Mimosoideae* | Subfamily | 6 | 0.06 | 906 | <0.01 |
| **52** | ***Rhopalocarpus*** | Genus | 6 | 0.06 | 737 | <0.01 |
| **53** | ***Ziziphus mauritania*** | Species | 5 | 0.05 | 14345 | 0.01 |
| **54** | *Plantaginaceae* | Family | 5 | 0.05 | 4437 | <0.01 |
| **55** | *Burseraceae* | Family | 5 | 0.05 | 2427 | <0.01 |
| **56** | *Coffea* | Genus | 5 | 0.05 | 1791 | <0.01 |
| **57** | ***Phanerodiscus diospyroidea*** | Species | 5 | 0.05 | 1323 | <0.01 |
| **58** | *Annona senegalensis* | Species | 5 | 0.05 | 1258 | <0.01 |
| **59** | *Fabaceae* | Family | 5 | 0.05 | 1104 | <0.01 |
| **60** | *Noronhia* | Genus | 5 | 0.05 | 1040 | <0.01 |
| **61** | ***Syzygium*** | Genus | 5 | 0.05 | 1033 | <0.01 |
| **62** | ***Ocotea*** | Genus | 5 | 0.05 | 954 | <0.01 |
| **63** | ***Reissantia angustipetala*** | Species | 5 | 0.05 | 644 | <0.01 |
| **64** | *Bakerella* | Genus | 5 | 0.05 | 419 | <0.01 |
| **65** | ***Polyalthia*** | Genus | 5 | 0.05 | 379 | <0.01 |
| **66** | *Sapindaceae* | Family | 5 | 0.05 | 121 | <0.01 |
| **67** | *Moraceae* | Family | 4 | 0.04 | 32521 | 0.02 |
| **68** | *Diospyros* | Genus | 4 | 0.04 | 6484 | <0.01 |
| **69** | ***Plectaneia thouarsii*** | Species | 4 | 0.04 | 5879 | <0.01 |
| **70** | *Fabaceae* | Family | 4 | 0.04 | 3700 | <0.01 |
| **71** | *Grewia* | Genus | 4 | 0.04 | 3219 | <0.01 |
| **72** | ***Pithecellobium dulce*** | Species | 4 | 0.04 | 1394 | <0.01 |
| **73** | *Albizia* | Genus | 4 | 0.04 | 1298 | <0.01 |
| **74** | *Cedrelopsis* | Genus | 4 | 0.04 | 978 | <0.01 |
| **75** | *Millettieae* | Tribe | 4 | 0.04 | 925 | <0.01 |
| **76** | ***Arachis*** | Genus | 4 | 0.04 | 681 | <0.01 |
| **77** | *Convolvulaceae* | Family | 4 | 0.04 | 647 | <0.01 |
| **78** | *Dichrostachys* | Genus | 4 | 0.04 | 555 | <0.01 |
| **79** | ***Solanum*** | Genus | 4 | 0.04 | 519 | <0.01 |
| **80** | *Sorindeia madagascariensis* | Species | 4 | 0.04 | 514 | <0.01 |
| **81** | *Apocynaceae* | Family | 3 | 0.03 | 11304 | 0.01 |
| **82** | ***Pittosporum*** | Genus | 3 | 0.03 | 9409 | 0.01 |
| **83** | ***Xeroscicyos*** | Genus | 3 | 0.03 | 4322 | <0.01 |
| **84** | *Drypetes* | Genus | 3 | 0.03 | 935 | <0.01 |
| **85** | *Mimosoideae* | Subfamily | 3 | 0.03 | 469 | <0.01 |
| **86** | ***Zanthoxylum*** | Genus | 3 | 0.03 | 468 | <0.01 |
| **87** | ***Hydrocotyle mannii*** | Species | 3 | 0.03 | 425 | <0.01 |
| **88** | *Moraceae* | Family | 3 | 0.03 | 353 | <0.01 |
| **89** | ***Dracaena*** | Genus | 3 | 0.03 | 250 | <0.01 |
| **90** | ***Erigeron naudinii*** | Species | 3 | 0.03 | 168 | <0.01 |
| **91** | *Foetidia* | Genus | 3 | 0.03 | 145 | <0.01 |
| **92** | *Bakerella* | Genus | 3 | 0.03 | 130 | <0.01 |
| **93** | ***Alantsilodendron villosum*** | Species | 3 | 0.03 | 118 | <0.01 |
| **94** | *Sapindaceae* | Family | 3 | 0.03 | 111 | <0.01 |
| **95** | ***Hyptis pectinata*** | Species | 2 | 0.02 | 7771 | <0.01 |
| **96** | ***Abrus*** | Genus | 2 | 0.02 | 4324 | <0.01 |
| **97** | *Baudouinia* | Genus | 2 | 0.02 | 2576 | <0.01 |
| **98** | ***Abrahamia*** | Genus | 2 | 0.02 | 516 | <0.01 |
| **99** | ***Grevea madagascariensis*** | Species | 2 | 0.02 | 432 | <0.01 |
| **100** | *Poeae* | Tribe | 2 | 0.02 | 340 | <0.01 |
| **101** | *Pooideae* | Subfamily | 2 | 0.02 | 286 | <0.01 |
| **102** | ***Salacia madagascariensis*** | Species | 2 | 0.02 | 172 | <0.01 |
| **103** | *Croton* | Genus | 2 | 0.02 | 144 | <0.01 |
| **104** | *Astereceae* | Family | 2 | 0.02 | 138 | <0.01 |
| **105** | *Albizia* | Genus | 1 | 0.01 | 4830 | <0.01 |
| **106** | ***Orzya*** | Genus | 1 | 0.01 | 1486 | <0.01 |
| **107** | ***Homalium*** | Genus | 1 | 0.01 | 1214 | <0.01 |
| **108** | *Myrtaceae* | Family | 1 | 0.01 | 1060 | <0.01 |
| **109** | ***Ormocarpum*** | Genus | 1 | 0.01 | 1053 | <0.01 |
| **110** | *Acacia* | Genus | 1 | 0.01 | 1036 | <0.01 |
| **111** | ***Brachylaena*** | Genus | 1 | 0.01 | 711 | <0.01 |
| **112** | ***Paullinia pinnata*** | Species | 1 | 0.01 | 674 | <0.01 |
| **113** | ***Rourea orientalis*** | Species | 1 | 0.01 | 430 | <0.01 |
| **114** | *Mimosoideae* | Subfamily | 1 | 0.01 | 381 | <0.01 |
| **115** | ***Alantsilodendron villosum*** | Species | 1 | 0.01 | 337 | <0.01 |
| **116** | *Fabaceae* | Family | 1 | 0.01 | 297 | <0.01 |
| **117** | *Poeae* | Tribe | 1 | 0.01 | 281 | <0.01 |
| **118** | *Brachylaena* | Genus | 1 | 0.01 | 264 | <0.01 |
| **119** | *Uncarina* | Genus | 1 | 0.01 | 229 | <0.01 |
| **120** | ***Barringtonia racemosa*** | Species | 1 | 0.01 | 225 | <0.01 |
| **121** | ***Gouania*** | Genus | 1 | 0.01 | 182 | <0.01 |
| **122** | ***Artabotrys*** | Genus | 1 | 0.01 | 145 | <0.01 |
| **123** | ***Oncostemum*** | Genus | 1 | 0.01 | 141 | <0.01 |
| **124** | ***Oncostemum*** | Genus | 1 | 0.01 | 137 | <0.01 |
| **125** | ***Crateva greveana*** | Species | 1 | 0.01 | 135 | <0.01 |
| **126** | *Oleaceae* | Family | 1 | 0.01 | 134 | <0.01 |
| **127** | *Bauhinia* | Genus | 1 | 0.01 | 132 | <0.01 |
| **128** | *Asteraceae* | Family | 1 | 0.01 | 124 | <0.01 |
| **129** | ***Maerua*** | Genus | 1 | 0.01 | 124 | <0.01 |
| **130** | *Araceae* | Family | 1 | 0.01 | 117 | <0.01 |
