# Supplementary material for: A DNA Metabarcoding Study of a Primate Dietary Diversity and Plasticity across Its Entire Fragmented Range
Source: PLoS One. 2013 Mar 19;8(3):e58971. doi: 10.1371/journal.pone.0058971 (PMC3602585; doi:10.1371/journal.pone.0058971)
Supplement: Table S2 — Canonical scores of the different MOTUs for the two first axes of the co-inertia analysis. (DOCX) [file pone.0058971.s002.docx]

**Table S2.** Canonical scores of the different MOTUs for the two first axes of the co-inertia analysis

| \| MOTU \| Taxa \| Axe1 \| \| --- \| --- \| --- \| \| ID042 \| *Meliaceae* \| -0.35 \| \| ID016 \| *Mimosoideae* \| -0.31 \| \| ID022 \| *Landolphia* \| -0.27 \| \| ID006 \| *Apocynaceae* \| -0.25 \| \| ID029 \| *Vigna* \| -0.25 \| \| ID045 \| *Melastomataceae* \| -0.24 \| \| ID017 \| *Ardisia* \| -0.23 \| \| ID039 \| *Cucurbitaceae* \| -0.15 \| \| ID113 \| *Terminalia* \| -0.13 \| \| ID058 \| *Dupuya* \| -0.13 \| \| ID159 \| *Anacardiaceae* \| -0.12 \| \| ID025 \| *Diospyros* \| -0.12 \| \| ID035 \| *Mimosoideae* \| -0.12 \| \| ID032 \| *Anacardium occidentale* \| 0.12 \| \| ID009 \| *Marsdenia* \| 0.13 \| \| ID044 \| *Trilepisium* \| 0.14 \| \| ID003 \| *Anacardiaceae* \| 0.14 \| \| ID021 \| *Ficus* \| 0.14 \| \| ID047 \| *Fabaceae* \| 0.17 \| \| ID013 \| *Mangifera indica* \| 0.26 \| | \| MOTU \| Taxa \| Axe2 \| \| --- \| --- \| --- \| \| ID020 \| *Rubiaceae* \| -0.28 \| \| ID083 \| *Cedrelopsis* \| -0.28 \| \| ID076 \| *Ipomoea* \| -0.27 \| \| ID012 \| *Neoapaloxylon madagascarensis* \| -0.24 \| \| ID017 \| *Ardisia* \| -0.24 \| \| ID194 \| *Mimosoideae* \| -0.24 \| \| ID054 \| *Burseraceae* \| -0.23 \| \| ID003 \| *Anacardiaceae* \| -0.19 \| \| ID015 \| *Sapotaceae* \| -0.18 \| \| ID195 \| *Zanthoxylum* \| -0.18 \| \| ID665 \| *Alantsilodendron vilosum* \| -0.18 \| \| ID113 \| *Terminalia* \| -0.14 \| \| ID596 \| *Bakerella* \| -0.13 \| \| ID033 \| *Solanum* \| -0.11 \| \| ID041 \| *Acacia* \| 0.10 \| \| ID029 \| *Vigna unguiculata* \| 0.10 \| \| ID005 \| *Cynometra* \| 0.11 \| \| ID047 \| *Fabaceae* \| 0.13 \| \| ID159 \| *Anacardiaceae* \| 0.13 \| \| ID011 \| *Sapindaceae* \| 0.14 \| \| ID025 \| *Diospyros* \| 0.15 \| \| ID004 \| *Olax* \| 0.20 \| \| ID006 \| *Apocynaceae* \| 0.21 \| |
| --- | --- | --- | --- | --- | --- | --- | --- | --- | --- | --- | --- | --- | --- | --- | --- | --- | --- | --- | --- | --- | --- | --- | --- | --- | --- | --- | --- | --- | --- | --- | --- | --- | --- | --- | --- | --- | --- | --- | --- | --- | --- | --- | --- | --- | --- | --- | --- | --- | --- | --- | --- | --- | --- | --- | --- | --- | --- | --- | --- | --- | --- | --- | --- | --- | --- | --- | --- | --- | --- | --- | --- | --- | --- | --- | --- | --- | --- | --- | --- | --- | --- | --- | --- | --- | --- | --- | --- | --- | --- | --- | --- | --- | --- | --- | --- | --- | --- | --- | --- | --- | --- | --- | --- | --- | --- | --- | --- | --- | --- | --- | --- | --- | --- | --- | --- | --- | --- | --- | --- | --- | --- | --- | --- | --- | --- | --- | --- | --- | --- | --- | --- | --- | --- | --- | --- | --- |
